# Mock trial as a simulation strategy allowing undergraduate nursing students to experience evidence-based practice: A scoping-review

**DOI:** 10.1371/journal.pone.0289789

**Published:** 2023-08-10

**Authors:** Chieun Song, Aeri Jang

**Affiliations:** Department of Nursing, Nambu University, Gwangju, South Korea; Iran University of Medical Sciences, ISLAMIC REPUBLIC OF IRAN

## Abstract

**Objective:**

The purpose of this scoping review was to determine the underlying design of simulations that help undergraduate nursing students acquire evidence-based practice (EBP) experiences.

**Method:**

The JBI methodology was used for this review. The inclusion criteria were studies conducted in academic, clinical, or virtual settings that examined simulation programs designed to facilitate the acquisition of EBP by undergraduate nursing students. A comprehensive search was performed on Jan 3, 2022, using the Medical Literature Analysis and Retrieval System Online (MEDLINE; PubMed), the Cumulative Index to Nursing and Allied Health Literature (CINAHL), the Education Resources Information Center (ERIC), and the Excerpta Medica database (EMBASE). Grey literature was not included. Publication year was limited to 2020 and later. There was no language restriction. Data were extracted using a tool developed by the reviewers and based on the National League for Nursing Jeffries Simulation Theory.

**Result:**

A total of 12,931 articles were found, and after duplicate articles and articles deemed ineligible based on the title and abstract (12,914 articles), 17 published papers were examined. The full texts of these studies were reviewed for eligibility, and one study was selected for the present scoping review. The selected study examined a mock trial designed to allow undergraduate nursing students to experience the ethical decision-making based on a diversity of evidence. The program reported in the study consisted of a prebriefing—simulation (mock)—debriefing structure with verified positive effects on EBP education.

**Conclusion:**

A mock trial is a useful educational strategy for allowing undergraduate nursing students to experience EBP, but a creative method should be found that can modify the mock trial for practical operation as the designing the program demands high levels of human and material resources.

**Registration:**

OSF Registries, https://osf.io/gdtyu, We updated OSF registry data for documenting important protocol amendments.

## Introduction

Evidence-based practice (EBP) is a decision-making process that considers clinical expertise, patients’ values, and the latest and most advanced evidence [1]. According to the American Association of Colleges of Nursing, it is essential for undergraduate nursing students to develop EBP competency prior to graduation [2]. Educators should consider ways to integrate EBP knowledge, skills, and attitudes into the curriculum [3]. If a new EBP course is not feasible, EBP content can be integrated into existing research methodology courses [4]. To increase students interest and participation, multidimensional strategies such as social media and games can be employed [5, 6]. Undergraduate nursing students recognize the need for EBP education and prefer trainings that allows for comparison of various EBP cases, rather than a single session as part of a major theory-based course [7].

Despite the importance of EBP, providing EBP education to undergraduate nursing students remains challenging due to the lack of human and material resources [8]. The organizational culture at universities and clinical training centers must be more supportive to EBP to facilitate its integration into clinical practice [1]. Finding a hospital that offers clinical training, let alone EBP training, is difficult, and finding qualified clinical educators is also a concern [9]. Additionally, the negative attitude of staff nurses and clinical educators toward EBP can cause confusion among students during clinical training [10]. An alternative approach is needed to help nursing education overcome these barriers and provide undergraduate nursing students with EBP experience.

Simulation is an effective educational strategy that creates a clinical setting for students to safely practice skills [11]. It enables students to gain knowledge, skills, and collaboration experience [12], reduces anxiety and enhances self-confidence [13], and develops clinical reasoning skills [14]. The inclusion of pre-briefing, simulation, and debriefing in the simulation’s design can impact student learning outcomes [15]. Thus, simulation is a valuable tool in nursing education that provides students with opportunities to connect contents with real-life situation [16]. In particular, simulation designs such as mock trials [17] are useful teaching and learning methods for students to experience how evidence-based practices are actually applied in clinical settings.

Previous scoping reviews of EBP education for undergraduate nursing students have focused on EBP educational intervention programs [18], barriers and facilitators in clinical education [10] and the use of interactive online technology for the EBP skill acquisition [19]. In situations where it is challenging to secure clinical practice settings with mature EBP cultures or clinical instructors possessing EBP competency, simulation is deemed a valuable teaching and learning approach for nursing students to acquire the experience of the EBP process. To attain the desired outcomes in EBP education, the development of simulation-based learning programs needs to be systematically conducted, and an exploration of how previous studies structured their simulations to facilitate the EBP process would be advantageous.

However, no scoping review has investigated simulations designed to help students experience EBP. This study aimed to fill that gap by collecting evidence to develop a simulation-based learning for undergraduate nursing students to experience EBP. The specific questions addressed in this study were 1) how are EBP simulations designed for undergraduate nursing students? and 2) what active learning strategies are used in these simulations?

## Materials and methods

The JBI methodology [20] was used to conduct this scoping review. The protocol of this study was registered in OSF registries and published [21]. All process of this scoping review were confirmed according to the Preferred Reporting Items for Systematic Reviews and Meta-Analyses for Scoping Reviews (PRISMA-Scr) [22]. The PRISMA ScR checklist is presented in S1 Checklist. In the first step, we derived research questions based on the Population, Concept, Context (PCC) framework. In the second step, we conducted a comprehensive search of relevant studies using four databases. The third step involved selecting literature based on predetermined inclusion and exclusion criteria. It is important to note that critical appraisal of the selected literature was not performed in this review. The reason for this is that the primary objective of our study was to examine how the simulation was designed to facilitate nursing students’ experiential learning in evidence-based practice, rather than providing specific answers regarding the effectiveness of the simulation. In the fourth step, we extracted information relevant to the research topic from the selected literature. Finally, in the fifth step, we analyzed and described the results. The specific methods employed in each step are outlined below:

### Eligibility criteria

#### Participants

In the scoping review, we selected studies in which the participants included nursing students in simulation programs. Studies of simulation programs for graduate or practicing nurses that did not involve undergraduate nursing students were excluded. Studies of inter-professional simulations for the cultivation of non-technical skills were also excluded.

#### Concept

The concept of this scoping review was to understand the design components of simulations that enable undergraduate nursing students to experience evidence-based practice process. Studies describing the strategies for EBP implementation in a simulation program were included in this review. Studies developing tools for evaluating simulations were excluded.

#### Context

In this review, we included studies in which simulation programs were presented in all settings, such as academic, clinical, or virtual settings. Studies related to scenario development and where simulation-based learning for simple knowledge transfer independent of EBP were excluded.

#### Types of sources

In this scoping review, study design was not a limiting factor. We did exclude review, opinion, or editorial articles.

#### Search strategy

This review was conducted according to a published protocol [21]. Following the full search strategy of the protocol, the literature search was performed on Jan 3, 2022. The publication year was restricted to include only papers published after 2000, but language was not restricted. Grey literature was excluded. The searched databases were MEDLINE, CINAHL, the Education Resources Information Center (ERIC), and the Excerpta Medica database (EMBASE). The search strategy for each database is presented in S1 Table.

#### Study/Source of evidence selection

**Fig 1:**
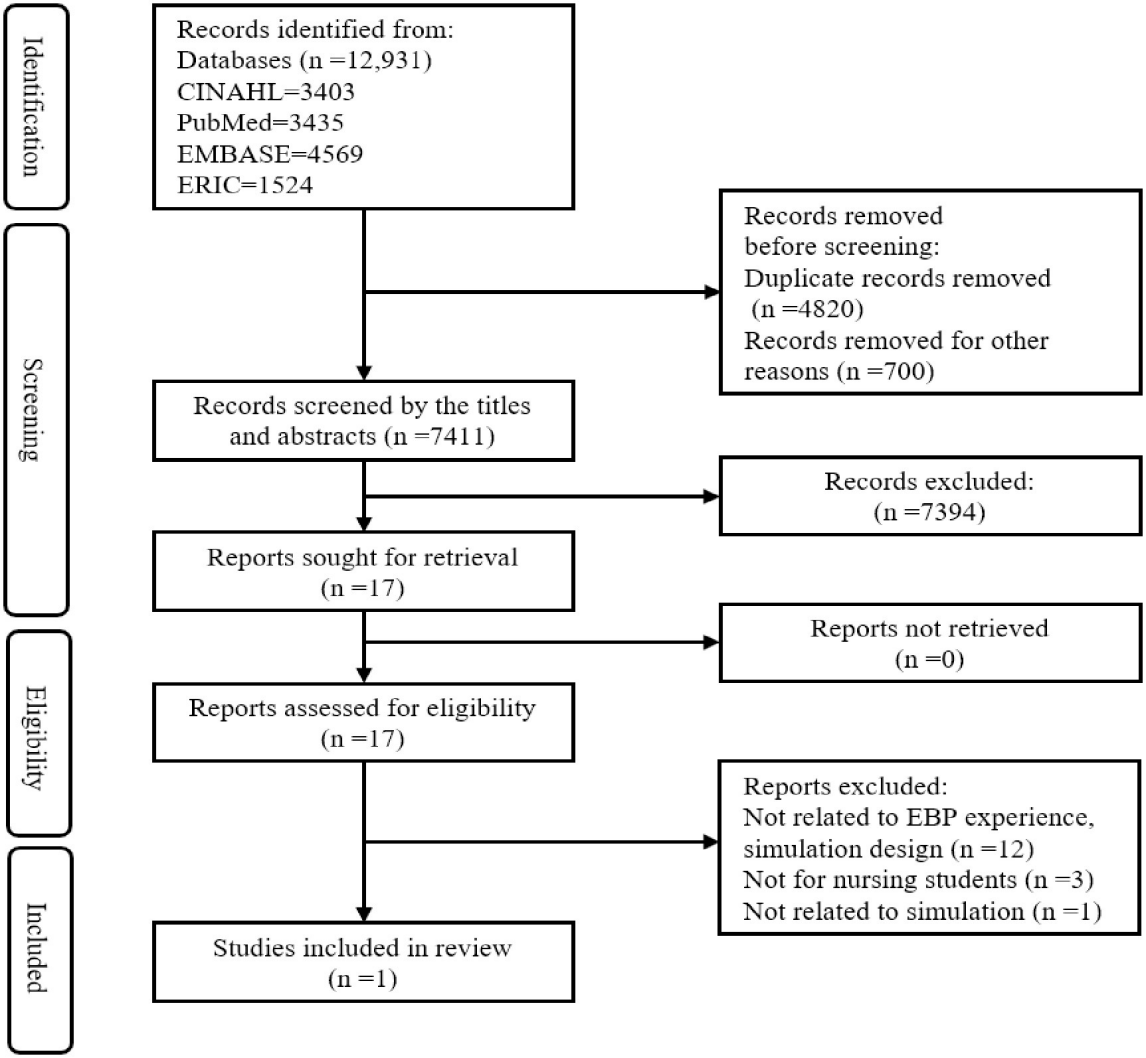
Flow diagram for the scoping review.

All identified citation were collated and uploaded into EndNote X8 8.2 (Clarivate Analytics, Philadelphia, PA, USA), and duplicates were removed. From the information presented in Endnote, if the reference style was not a journal type, it was removed. The first author screened the titles and abstracts and assessed them according to the review criteria, then removed ineligible citations. The full text of the selected citations was assessed in detail by two independent reviewers. There was no disagreement between the reviewers in the selection process. The reasons for exclusion of full-text articles were presented in flow diagram (Fig 1).

#### Data extraction, synthesis and analysis

Data was extracted by the first author using a data-extraction tool developed based on the NLN Jeffries Simulation Theory [23], which was widely used in simulation-based education [24] and provided a clear framework for identifying the key components of a simulation and how they interact. Extracted data was verified by the second author.

The extracted data included the authors, year of publication, and country of origin, context factor, the goals of the simulation, the curriculum fit, the specific learning objectives, fidelity, briefing/debriefing strategies, outcomes, and key findings relevant to the review question. A pilot review was included in the protocol, which was omitted as only a single article was selected. There was no disagreement between the reviewers in the data extraction process.

The data was presented in narrative summary according to core elements of the NLN Jeffries Simulation Theory to describe the characteristics of the simulation design.

#### Ethical consideration

This scoping review was exempted from approval by the Institutional Review Board of Nambu University, Gwangju, South Korea (1041478-2021-HR-029).

## Results

We searched 12931 citations and removed 4820 duplicates and 700 articles of non-journal type. Seventeen articles were selected by screening the titles and abstracts and two independent reviewers assessed the full text in detail. Finally, one article was included for this review (Fig 1).

The final article selected for the present scoping review was a mock trial study designed to enable students to experience the process of evidence-based ethical decision-making in clinical practice, as conducted by Opsahl et al. [17]. The list of included and excluded studies are presented in S1 File.

The study reported on the experience of inter-professional education through the development and operation of a mock hospital ethics committee (MHEC) by the faculty members of the departments of nursing, social welfare, and law at a university located in a mid-western region of the U.S. A total of 121 students from the departments of nursing, social welfare, law and medicine participated in the program and data from 80 undergraduate nursing students were used in the qualitative analysis.

### Context & background

The study was designed for an educational purpose to give undergraduate students a direct experience of how various pieces of evidence might be applied in ethical decision-making. To provide a more realistic experience, the mock trial was conducted in an auditorium at a local hospital. The MHEC was part of the students’ ethics class coursework, but the year in school, or the distribution of years in school, for participating students was unclear. The program consisted of two scenarios conducted for 2.5 hr.

### Simulation design

The learning objectives of the MHEC suggested in Opsahl et al. [17] were to enable students to recognize the need for and process of ethics review and analyze the ethical dilemmas in clinical practice so they could make sound inferences based on ethical principles, and to comprehend the varying forms of evidence that could be applied in ethical decision-making. They also needed to understand the importance of the inter-professional committee and identifying the contextual factors that could influence ethical decision-making and optimal outcomes.

They used the pre-briefing strategies to help students learn the main concepts of ethical principles and EBP. They also used the assignment to analyze a case study related to ethical decision-making to enable students to seek evidence, apply the ethical principles, and identify the influencing contextual factors of decision-making independently.

As with actual ethical committees at hospitals, the participating members of the MHEC were nurses, doctors, lawyers, pharmacists, Catholic priests, and social workers. Each scenario comprised 10 min introduction, 10 min scenario review, 20 min discussion, 30 min debriefing and 10 min rest before the next scenario. In the mock trial, the students played the role of observers. The debriefing systematically proceeded using the Debriefing for Meaningful Learning method, with discussions and interactions among the MHEC members and students. Afterwards, the students submitted a report reanalyzing the scenario from their previous assignment.

### Outcomes

In Opsahl et al. [17], the outcome measure was the knowledge gain by undergraduate nursing students. For this, the post-MHEC analysis reports were evaluated through thematic content analysis and the findings were reported. The MHEC was shown to have helped nursing students acquire conceptual knowledge regarding ethics, group dynamics and discipline-specific responsibility. The MHEC was also shown to have motivated the students to recognize the need for varying forms of evidence in ethical decision-making and the importance of evidence sharing across multidisciplinary professionals.

## Discussion

This scoping review was conducted to explore how simulation education was designed to provide undergraduate nursing students with an experience of the EBP process. While the review initially planned to extensively review papers selected based on the core elements of the NLN Jeffries Simulation Theory, only a single paper was ultimately selected for inclusion. Despite this limitation, our review provides valuable insights into the use of mock trials as a strategy for simulation-based EBP education. Our finding suggest that mock trial may be an effective way to engage students in the EBP process by providing opportunities to seek evidence, apply ethical principles and identify contextual factors that influence decision-making. However, further research is needed to validate these findings and explore the potential benefits of other simulation-based strategies for EBP education.

Based on this scoping review, we discussed the factors relevant to utilizing a mock trial as a strategy for a simulation of EBP education.

First, the selection of a suitable theme is important. The selected theme should be appropriate for the purpose of education and in line with participants’ interests. In Opsahl et al. [17], the theme of the scenario was selected to present diverse evidence drawn from each participant’s specialty and using a multidisciplinary educational program for the evidence-based ethical decision-making. Students from social welfare, law, and medicine all participated along with the nursing students. In mock trials targeting clinical nurses, the selected theme was one that could draw interest from all participants, including those who were nurses from different hospitals or departments [25, 26]. In mock trials during undergraduate education, it could be useful to collect theoretical issues raised by the students who had clinical training experience and use those issues to create the mock trial’s theme.

Much time and effort are required to prepare for this type of simulation, including the script write-up, subject selection, and rehearsal. Scheduling sufficient time prior to running the mock trial is therefore predicted to be important. In the report of Opsahl et al. [17], although the actual length of preparation was not clearly stated, it could be conjectured that considerable time and effort were spent in recruiting experts from various fields, finding a suitable location for the mock trial, writing the script to present varying forms of evidence and performing the rehearsal, all to ensure that the mock trial’s design could simulate an actual MHEC. In Cetrella-Nigro et al. [25], approximately four months were spent in preparation, with two months solely spent on script writing. Another challenge was to recruit all actors for the rehearsal; it was also necessary to effectively convey accurate information to educators using materials such as PowerPoint in addition to verbal explanation. In Staffileno [26], the reported preparation time was approximately three months. Efforts were taken to ensure the composition of a valid and reliable scenario by involving all participants of the mock trial in conducting the literature review and taking time to discuss their findings. In addition, plans for recording the process should be made to convey the education to those who do not participate in the mock trial [25, 26].

In designing a mock trial, decisions should be made about how to induce students’ participation as they play the role of observers to ensure they acquire the experience of EBP. In Opsahl et al. [17], a 30 min debriefing session was included in the design to allow the observer students and the mock trial members to discuss the session and interact. In addition, the scenario was released to everyone before the mock trial. Through assignments, participants were guided to write up an analysis report so they could be fully aware of the relevant contents at the mock trial. In Centrella-Nigro et al. [25], the observers were given an opportunity to vote before the final decision was made after running the scenario.

Lastly, as much time and effort are required to design a typical original mock trial, a creative method should be sought to modify the mock trial design for effective operation within a limited educational period. Previous studies showed creative applications of the mock trial method of EBP education, where a selected set of students as the mock panel held discussions in a PhD course [27] or a PhD student made a presentation in an undergraduate class [28]. In addition, since running a mock trial as regular coursework would place a high burden on the educator, a method should be developed to encourage student participation in mock trials run at hospitals [25] or to provide it as part of a non-regular course, such as a mock trial club activity.

The limitations of this scoping review are as follows.

In this review, considering that EBP began to be emphasized in nursing education in early 2000s [29], the year of publication was limited to 2000 or later. As this review method does not assess the quality of selected studies, grey literature that has not undergone a peer review process was excluded to ensure the quality and reliability of included studies. Therefore, all literature related to the topic of this study may not have been included in the evidence selection process. Considering that only one study was identified for inclusion, the implications of this limitation should also be explored. The review raises important questions regarding the extent of evidence available for simulation-based EBP education in the nursing context, and highlights gaps in current knowledge. Further research is needed to address these gaps and to explore how simulation can be effectively incorporated into nursing education and training. This review could serve as a basis for generating hypotheses and developing more specific systematic reviews in the future.

Overall, the contribution of this review to the field of nursing education and training lies in its identification of the current state of evidence on the use of simulation for EBP education. While the limitations of the review must be taken into account, the findings provide valuable insights for educators and researchers in the field.

## Conclusions

For undergraduate nursing students, EBP experience may help them improve their self-efficacy regarding the practice and help encourage them to be early adopters regarding the implementation of EBP as future nurses. A mock trial is considered an effective educational strategy to help both nurses and nursing students acquire direct experience of how evidence is used to make clinical decisions. However, as mock trial preparation demands significant time and effort, creative strategies should be developed to effectively and efficiently create them, such as establishing liaisons across hospitals and universities or pursuing integrated education across graduate and undergraduate courses. In addition, further studies should examine the simulation-based educational programs that build EBP experience in nurses and varying strategies for simulation-based EBP education.

## Supporting information

S1 Checklist PRISMA ScR checklist. (PDF)

S1 Table Search strategy. (DOCX)

S1 File List of included and excluded studies. (DOCX)

## References

[1] Melnyk BM, Fineout-Overholt E. Evidence-based practice in nursing & healthcare: A guide to best practice. Netherlands: Wolters Kluwer; 2019.

[2] American Association of Colleges of Nursing 2021. The essentials: Core competencies for professional nursing education. Washington, DC: American Association of Colleges of Nursing; 2021.

[3] Kerr H, Rainey D. Addressing the current challenges of adopting evidence-based practice in nursing. Br J Nurs. 2021;30(16):970–974. doi: 10.12968/bjon.2021.30.16.970 34514831

[4] Song CE, Park H, Lee M, Stevens KR. Integrating EBP into an undergraduate research methodology course using the Star Model of Knowledge Transformation: A mixed-method study. Nurse Educ Today. 2019;105:105021. doi: 10.1016/j.nedt.2021.105021 34214950

[5] Milner KA, McCloud R, Cullen J. The evidence appraisal game: An innovative strategy for teaching step 3 in evidence-based practice. Worldviews Evid Based Nurs. 2020;17(2):173–175. doi: 10.1111/wvn.12433 32233017

[6] Song CE, Park H. Active learning in e-learning programs for evidence-based nursing in academic settings: A scoping review. J Contin Educ Nurs. 2021;52(9):407–412. doi: 10.3928/00220124-20210804-05 34432581

[7] Song C, Kim W, Park J. What should be considered in the evidence-based practice competency-based curriculum for undergraduate nursing students? From the student’s point of view. Int J Environ Res Public Health. 2021;18(20):10965. doi: 10.3390/ijerph182010965 34682713 PMC8536150

[8] Duncombe DC. A multi-institutional study of the perceived barriers and facilitators to implementing evidence-based practice. J Clin Nurs. 2018;27(5–6):1216–1226. doi: 10.1111/jocn.14168 29149462

[9] Malik G, McKenna L, Plummer V. Facilitators and barriers to evidence-based practice: perceptions of nurse educators, clinical coaches and nurse specialists from a descriptive study. Contemp Nurse. 2016;52(5):544–554. doi: 10.1080/10376178.2016.1188017 27160348

[10] Fiset VJ, Graham ID, Davies BL. Evidence-based practice in clinical nursing education: A scoping review. J Nurs Educ. 2017;56(9):534–541. doi: 10.3928/01484834-20170817-04 28876439

[11] Bogossian FE, Cant RP, Ballard EL, Cooper SJ, Levett-Jones TL, McKenna LG, et al. Locating "gold standard" evidence for simulation as a substitute for clinical practice in prelicensure health professional education: A systematic review. J Clin Nurs. 2019;28(21–22):3759–3775. doi: 10.1111/jocn.14965 31216367

[12] Li YY, Au ML, Tong LK, Ng WI, Wang SC. High-fidelity simulation in undergraduate nursing education: A meta-analysis. Nurse Educ Today. 2022;111:105291. doi: 10.1016/j.nedt.2022.105291 35158134

[13] Labrague LJ, McEnroe-Petitte DM, Bowling AM, Nwafor CE, Tsaras K. High-fidelity simulation and nursing students’ anxiety and self-confidence: A systematic review. Nurs Forum. 2019;54(3):358–368. doi: 10.1111/nuf.12337 30852844

[14] Theobald KA, Tutticci N, Ramsbotham J, Johnston S. Effectiveness of using simulation in the development of clinical reasoning in undergraduate nursing students: A systematic review. Nurse Educ Pract. 2021;57:103220. doi: 10.1016/j.nepr.2021.103220 34781195

[15] Tong LK, Li YY, Au ML, Wang SC, Ng WI. High-fidelity simulation duration and learning outcomes among undergraduate nursing students: A systematic review and meta-analysis. Nurse Educ Today. 2022;116:105435. doi: 10.1016/j.nedt.2022.105435 35728333

[16] Sofer D. The Value of Simulation in Nursing Education. Am J Nurs. 2018;118(4):17–18. doi: 10.1097/01.NAJ.0000532063.79102.19 29596245

[17] Opsahl JA, Nelson T, Madeira J, Wonder AH. Evidence-based, ethical decision-making: Using simulation to teach the application of evidence and ethics in practice. Worldviews Evid Based Nurs. 2020;17(6):412–417. doi: 10.1111/wvn.12465 33001572

[18] Patelarou AE, Mechili EA, Ruzafa-Martinez M, Dolezel J, Gotlib J, Skela-Savič B, et al. Educational interventions for teaching evidence-based practice to undergraduate nursing students: A scoping review. Int J Environ Res Public Health. 2020;17(17):6351. doi: 10.3390/ijerph17176351 32878256 PMC7503534

[19] Shorey S, Chua JYX. Nursing students’ insights of learning evidence-based practice skills using interactive online technology: Scoping review. Nurs Health Sci. 2022;24(1):83–92. doi: 10.1111/nhs.12915 34923735

[20] McInerney P, Munn Z, Tricco AC, Khalil H. Chapter 11. Peters MDJ GC. In Aromataris E, Munn Z, editors. JBI Manual for Evidence Synthesis, JBI. https://synthesismanual.jbi.global: Scoping Reviews (2020 version). 2020; 2020.

[21] Song CE, Jang A. Simulation design for improvement of undergraduate nursing students’ experience of evidence-based practice: A scoping-review protocol. PLoS One. 2021;16(11):e0260238. doi: 10.1371/journal.pone.0260238 34793579 PMC8601474

[22] Tricco AC, Lillie E, Zarin W, O’Brien KK, Colquhoun H, Levac D, et al. PRISMA Extension for Scoping Reviews (PRISMA-ScR): Checklist and Explanation. Ann Intern Med. 2018;169(7): 467–473. doi: 10.7326/M18-0850 . 30178033

[23] Jeffries PR. The NLN Jeffries Simulation Theory. 2nd ed. National League for Nursing. Philadelphia:Wolters Kluwer; 2022.

[24] Lavoie P, Michaud C, Belisle M, Boyer L, Gosselin E, Grondin M, et al. Learning theories and tools for the assessment of core nursing competencies in simulation: A theoretical review. J Adv Nurs. 2018;74(2): 239–250. doi: 10.1111/jan.13416 28815750

[25] Centrella-Nigro AM, Flynn D. Teaching evidence-based practice using a mock trial. J Contin Educ Nurs. 2012;43(12):566–70. doi: 10.3928/00220124-20120917-27 22998038

[26] Staffileno BA, McKinney C. Utilizing a mock trial to demonstrate evidence-based nursing practice: a staff development process. J Nurses Staff Dev. 2010;26(2):73–6. doi: 10.1097/NND.0b013e3181d47aab 20354408

[27] Moralis IF, Cassiano A, Medeiros SM, Menezes R, Dantas R, Dantas DV, et al. Mock panels as an active teaching methodology in the education of nursing doctors. Revista Brasileira de Enfermagem, 2020;73(6):e20190700. doi: 10.1590/0034-7167-2019-0700 32901751

[28] Reynolds SS. Mock nursing research and evidence-based practice conference to support learning in pre-licensure nursing students. Worldviews Evid Based Nurs. 2019;16(6):498–500. doi: 10.1111/wvn.12412 31721417

[29] Stevens KR, Staley JM. The Quality Chasm reports, evidence-based practice, and nursing’s response to improve healthcare. Nurs Outlook. 2006;54(2):94–101. doi: 10.1016/j.outlook.2005.11.007 16597528

