# Supplementary material for: Mock trial as a simulation strategy allowing undergraduate nursing students to experience evidence-based practice: A scoping-review
Source: PLoS One. 2023 Aug 10;18(8):e0289789. doi: 10.1371/journal.pone.0289789 (PMC10414593; doi:10.1371/journal.pone.0289789)
Supplement: S1 Table — (DOCX) [file pone.0289789.s002.docx]

1. Search strategy for MEDLINE (PubMed) (2022_1_3)

| **#** | **query** |  |
| --- | --- | --- |
| 1 | (education, nursing, baccalaureate[MeSH Terms]) OR (students, nursing[MeSH Terms]) | 38,078 |
| 2 | "nursing student*"[Title/Abstract] OR "undergraduate"[Title/Abstract] OR "pre-registration nursing"[Title/Abstract] OR "baccalaureate"[Title/Abstract] OR "Nursing"[Title/Abstract] | 326,180 |
| 3 | #1 OR #2 | 339,094 |
| 4 | "Computer Simulation"[MeSH Terms] OR "Patient Simulation"[MeSH Terms] OR "High Fidelity Simulation Training"[MeSH Terms] OR "Simulation Training"[MeSH Terms] OR "Virtual Reality"[MeSH Terms] | 278,353 |
| 5 | "simulat*"[Title/Abstract] OR "virtual"[Title/Abstract] OR "high-fidelity"[Title/Abstract] OR "manikins"[Title/Abstract] | 674,095 |
| 6 | #4 OR #5 | 805,558 |
| 7 | "Evidence-Based Practice"[MeSH Terms] OR "Evidence-Based Nursing"[MeSH Terms] | 92,318 |
| 8 | "evidence"[Title/Abstract] OR "evidence-based"[Title/Abstract] OR "appraisal"[Title/Abstract] OR "application"[Title/Abstract] OR "EBP"[Title/Abstract] OR "inquiry"[Title/Abstract] OR "clinical question*"[Title/Abstract] OR "decision-making"[Title/Abstract] OR "guideline*"[Title/Abstract] OR "research"[Title/Abstract] OR "management"[Title/Abstract] | 5,563,375 |
| 9 | #7 OR #8 | 5,587,772 |
| 10 | #3 AND #6 AND #9; Filters: from 2000 | 3435 |

1. Search strategy for CINAHL (2022_1_3)

| **#** | **query** |  |
| --- | --- | --- |
| 1 | (MM "Students, Nursing+") OR (MM "Students, Nursing, Baccalaureate+") | 27,422 |
| 2 | "nursing student*"[Title/Abstract] OR "undergraduate"[Title/Abstract] OR "pre-registration nursing"[Title/Abstract] OR "baccalaureate"[Title/Abstract] OR "nursing"[Title/Abstract] | 350,427 |
| 3 | #1 OR #2 | 358,376 |
| 4 | (MM "Computer Simulation+") OR (MM "Simulations+") OR (MM "Patient Simulation") | 19,794 |
| 5 | "simulat*"[Title/Abstract] OR "virtual"[Title/Abstract] OR "high-fidelity"[Title/Abstract] OR "manikins"[Title/Abstract] | 91,468 |
| 6 | #4 OR #5 | 95,640 |
| 7 | (MM "Nursing Practice, Evidence-Based+") OR (MM "Nursing Practice, Research-Based") OR (MM "Education, Nursing, Research-Based") | 10,036 |
| 8 | "evidence"[Title/Abstract] OR "evidence-based"[Title/Abstract] OR "appraisal"[Title/Abstract] OR "application"[Title/Abstract] OR "EBP"[Title/Abstract] OR "inquiry"[Title/Abstract] OR "clinical question*"[Title/Abstract] OR "decision-making"[Title/Abstract] OR "guideline*"[Title/Abstract] OR "research"[Title/Abstract] OR "management"[Title/Abstract] | 1,607,354 |
| 9 | #7 OR #8 | 1,614,763 |
| 10 | #3 AND #6 AND #9; Filters: from 2000 | 3403 |

1. Search strategy for EMBASE (2022_1_3)

| **#** | **query** |  |
| --- | --- | --- |
| 1 | ‘nursing student’/exp | 29,773 |
| 2 | "nursing student*"[Title/Abstract] OR "undergraduate"[Title/Abstract] OR "pre-registration nursing"[Title/Abstract] OR "baccalaureate"[Title/Abstract] OR "nursing"[Title/Abstract] | 357,191 |
| 3 | #1 OR #2 | 366,611 |
| 4 | ‘Computer Simulation’/exp OR ‘Simulations’/exp OR ‘Patient Simulation’/exp | 374,260 |
| 5 | "simulat*"[Title/Abstract] OR "virtual"[Title/Abstract] OR "high-fidelity"[Title/Abstract] OR "manikins"[Title/Abstract] | 726,860 |
| 6 | #4 OR #5 | 819,101 |
| 7 | ‘evidence based practice’/exp OR ‘evidence based nursing’/exp | 1,527,555 |
| 8 | "evidence"[Title/Abstract] OR "evidence-based"[Title/Abstract] OR "appraisal"[Title/Abstract] OR "application"[Title/Abstract] OR "EBP"[Title/Abstract] OR "inquiry"[Title/Abstract] OR "clinical question*"[Title/Abstract] OR "decision-making"[Title/Abstract] OR "guideline*"[Title/Abstract] OR "research"[Title/Abstract] OR "management"[Title/Abstract] | 7,131,400 |
| 9 | #7 OR #8 | 8,036,495 |
| 10 | #3 AND #6 AND #9; Filters: from 2000 | 4569 |

1. Search strategy for ERIC (2022_1_3)

| **#** | **query** |  |
| --- | --- | --- |
|  | ("nursing student*" OR "undergraduate" OR "pre-registration nursing" OR "baccalaureate" OR "nursing") AND ("simulat*" OR "virtual" OR "high-fidelity" OR "manikins") AND ("evidence" OR "evidence-based" OR "appraisal" OR "application" OR "EBP" OR "inquiry" OR "clinical question*" OR "decision-making" OR "guideline*" OR "research" OR "management")  ; Filters: from 2000 | 1524 |
