# Supplementary material for: Mock trial as a simulation strategy allowing undergraduate nursing students to experience evidence-based practice: A scoping-review
Source: PLoS One. 2023 Aug 10;18(8):e0289789. doi: 10.1371/journal.pone.0289789 (PMC10414593; doi:10.1371/journal.pone.0289789)
Supplement: S1 File — (DOCX) [file pone.0289789.s003.docx]

**List of included and excluded studies**

■ Included study

1. Opsahl A, Nelson T, Madeira J, Wonder AH. Evidence-Based, Ethical Decision-Making: Using Simulation to Teach the Application of Evidence and Ethics in Practice. Worldviews on evidence-based nursing. 2020;17(6):412-7.

■ Excluded study

1. Guhde J. Using online exercises and patient simulation to improve students' clinical decision-making. Nursing education perspectives. 2010;31(6):387-9.

2. Rababa M, Masha'al D. Using branching path simulations in critical thinking of pain management among nursing students: Experimental study. Nurse education today. 2020;86:104323.

3. White CT. Using a Mock Trial Method to Enhance Effectiveness of Teaching Evidence-Based Practice in Nursing. Journal for nurses in professional development. 2015;31(6):E11-E4.

4. Burbach B, Barnason S, Thompson SA. Using "think aloud" to capture clinical reasoning during patient simulation. International journal of nursing education scholarship. 2015;12.

5. Gabriel PM, Lieb CL, Holland S, Ballinghoff J, Cacchione PZ, McPeake L. Teaching Evidence-Based Sepsis Care: A Sepsis Escape Room. Journal of continuing education in nursing. 2021;52(5):217-25.

6. Centrella-Nigro AM, Flynn D. Teaching Evidence-Based Practice Using a Mock Trial. Journal of Continuing Education in Nursing. 2012;43(12):566-70.

7. Raurell-Torredà M, Romero-Collado À. Simulation-Based Learning as a Tactic for Teaching Evidence-Based Practice. Worldviews on evidence-based nursing / Sigma Theta Tau International, Honor Society of Nursing. 2015;12(6):392-4.

8. Farrar FC, Suggs L. Empowering Critical Thinking Skills with Computerized Patient Simulators. Journal of College Teaching & Learning. 2010;7(5):1-4.

9. Şahin G, Başak T. The Effect of Virtual Patient Simulation on Nursing Students' Clinical Decision Making and Problem-Solving Skills. Journal of Education & Research in Nursing / Hemsirelikte Egitim ve Arastirma Dergisi. 2021;18(2):178-82.

10. Rigby L, Wilson I, Baker J, Walton T, Price O, Dunne K, et al. The development and evaluation of a 'blended' enquiry based learning model for mental health nursing students: "making your experience count". Nurse Education Today. 2012;32(3):303-8.

11. Beattie B, Koroll D, Price S. Designing Nursing Simulation Clinical Experiences to Promote Critical Inquiry. College Quarterly. 2010;13(1).

12. Zarifsanaiey N, Amini M, Saadat F. A comparison of educational strategies for the acquisition of nursing student's performance and critical thinking: simulation-based training vs. integrated training (simulation and critical thinking strategies). BMC medical education. 2016;16(1):294.

13. Dillard N, Sideras S, Ryan M, Hodson KC, Lasater K, Siktberg L. A collaborative project to apply and evaluate the clinical judgment Model through simulation. Nursing Education Perspectives. 2009;30(2):99-104.

14. Goodstone L, Cherkis F, Glaser C, Nikolaidou M, Maggio NJ. Bundle up: Introducing care bundles to increase knowledge and confidence of senior nursing students. Teaching & Learning in Nursing. 2015;10(3):143-8.

15. Mitchell ML, Henderson A, Jeffrey C, Nulty D, Groves M, Kelly M, et al. Application of best practice guidelines for OSCEs-An Australian evaluation of their feasibility and value. Nurse education today. 2015;35(5):700-5.

16. Ross JG, Burrell SA. Standardized Patient Simulation to Facilitate Learning in Evidence-Based Oncology Symptom Management. The Journal of nursing education. 2018;57(4):250-3.
